# Resistance to Oncolytic Myxoma Virus Therapy in Nf1^−/−^/Trp53^−/−^ Syngeneic Mouse Glioma Models Is Independent of Anti-Viral Type-I Interferon

**DOI:** 10.1371/journal.pone.0065801

**Published:** 2013-06-06

**Authors:** Franz J. Zemp, Brienne A. McKenzie, Xueqing Lun, Lori Maxwell, Karlyne M. Reilly, Grant McFadden, V. Wee Yong, Peter A. Forsyth

**Affiliations:** 1 Department of Oncology, Clark H. Smith Brain Tumor Center, University of Calgary, Tom Baker Cancer Centre, Southern Alberta Cancer Research Institute, Calgary, Alberta, Canada; 2 Clark H. Smith Brain Tumor Center, University of Calgary, Alberta, Canada; 3 Mouse Cancer Genetics Program, National Cancer Institute, Frederick, Maryland, United States of America; 4 Department of Molecular Genetics and Microbiology, College of Medicine, University of Florida, Gainesville, Florida, United States of America; 5 Departments of Clinical Neurosciences and Oncology, Hotchkiss Brain Institute, University of Calgary, Alberta, Canada; 6 Moffitt Cancer Center and Research Institute and University of Southern Florida, Tampa, Florida, United States of America; University of Chicago, United States of America

## Abstract

Despite promising preclinical studies, oncolytic viral therapy for malignant gliomas has resulted in variable, but underwhelming results in clinical evaluations. Of concern are the low levels of tumour infection and viral replication within the tumour. This discrepancy between the laboratory and the clinic could result from the disparity of xenograft versus syngeneic models in determining *in vivo* viral infection, replication and treatment efficacy. Here we describe a panel of primary mouse glioma lines derived from *Nf1* ^+/−^ *Trp53* ^+/−^ mice in the C57Bl/6J background for use in the preclinical testing of the oncolytic virus Myxoma (**MYXV**). These lines show a range of susceptibility to MYXV replication *in vitro*, but all succumb to viral-mediated cell death. Two of these lines orthotopically grafted produced aggressive gliomas. Intracranial injection of MYXV failed to result in sustained viral replication or treatment efficacy, with minimal tumour infection that was completely resolved by 7 days post-infection. We hypothesized that the stromal production of Type-I interferons (**IFNα/β**) could explain the resistance seen in these models; however, we found that neither the cell lines *in vitro* nor the tumours *in vivo* produce any IFNα/β in response to MYXV infection. To confirm IFNα/β did not play a role in this resistance, we ablated the ability of tumours to respond to IFNα/β via IRF9 knockdown, and generated identical results. Our studies demonstrate that these syngeneic cell lines are relevant preclinical models for testing experimental glioma treatments, and show that IFNα/β is not responsible for the MYXV treatment resistance seen in syngeneic glioma models.

## Introduction

Malignant gliomas (**MGs**) are devastating diseases and, although relatively rare, are almost universally terminal. The most severe, Grade IV gliomas (glioblastoma multiforme), have a median survival of only 12–15 months [1], [2], a prognosis which has seen little improvement over several decades [3]. The obvious need for new therapies has fostered a number of experimental treatment options, including the use of oncolytic viruses.

Oncolytic virus (**OV**) therapy entails the use of replication competent viruses that selectively infect and kill cancer cells, as well as potentially initiate anti-tumoural immune responses. One of the most appealing tenants of OV therapy is the treatment's self-potentiation, which hinges on the infection, replication, and spread of the virus throughout the tumor. Several OVs have been evaluated in MG clinical trials [4], [5], and although safe, only a handful of clinical responses have been observed and evidence of tumour infection and viral replication was limited to very few patients [6], [7]. A likely explanation for the poor and variable clinical outcome despite promising preclinical results is the extensive reliance on xenograft glioma models for OV preclinical evaluation. Xenografts possess two important, but potentially overlapping limitations that compromise their utility as preclinical OV models. Firstly, the immunocompromised nature of these models inevitably affects the profile of immune infiltrates within the tumour microenvironment. By changing the cellular components of the tumour stroma, the cytokine, chemokine, and growth factor networks, which may be vitally important in OV resistance mechanisms, are fundamentally changed. Secondly, the antiviral signalling networks that exist within the murine tumour stroma may not signal effectively to the human tumour due to inter-species receptor-ligand incompatibility. Xenografts thus ignore many potential interactions between the glioma and its microenvironment prior or during OV treatment. These interactions are potentially of great importance, especially when considering potential immune-glioma interactions that could occur when using a replication-competent virus.

We have previously found differences between xenograft and syngeneic MG models using Myxoma virus (**MYXV**), where MYXV has robust viral replication and often cures immunocompromised mice bearing orthotopic brain-tumor xenografts [8], [9], but does not have comparable replication or efficacy in syngeneic rodent MGs [10]. This is in dramatic contrast to tumour models outside the brain, such as disseminated pancreatic cancer in the intraperitoneal cavity, where MYXV virotherapy is most effective in syngeneic models where the full spectrum of innate and acquired immune responses were intact [11], [12]. The OV treatment resistance we have seen for MG in immunocompetent models is worrisome, as the syngeneic tumours used strongly recapitulate human MGs [13] and retain immune cell populations and stromal interactions that are expected in MG patients. In order to interrogate the immune contributions that may affect efficacy of OVs in MGs, relevant murine MG models in the C57Bl/6 background are ideal, as this background has numerous mutant and transgenic strains that could assist in delineating these mechanisms.

Here we characterize a panel of primary mouse glioma cell lines for the preclinical testing of oncolytic viral therapy, including the orthotopic grafting of these lines into C57Bl/6J mice. These lines were derived from C57Bl/6J NPcis mice (*Trp53* ^+/−^/*Nf1* ^+/−^), which spontaneously develop high-grade gliomas that recapitulate many clinical phenotypes of the human disease [14]–[16]. These lines show loss-of-heterozygosity of *Nf1* (encoding neurofibromin) and *Trp53* (encoding p53) in addition to PDGFRα expression [17], clinically relevant molecular features seen in many MG patients [18], [19]. Here we show that the NPcis lines show a range of susceptibility to MYXV replication *in vitro*, but all succumb to viral-mediated cell death. Two of these lines, K1492 and K1861, orthotopically grafted in C57Bl/6 mice produced aggressive tumours that retain human glioma markers. Despite their susceptibility to infection and replication *in vitro*, intracranial injection of MYXV failed to result in viral replication or treatment efficacy, with minimal tumour infection that was completely resolved by 7 days post-infection. These results closely mimic what we have previously shown in syngeneic rat models with MYXV [10], and has viral clearance kinetics similar to what others have shown in syngeneic mouse models with other OVs [20], [21]. We believe this to be an important and relevant resistance mechanism that must be overcome for OVs to reach maximal success in the clinic.

Type-I interferon (**IFNα/β**) is the quintessential anti-viral cytokine, being able to mediate anti-viral responses in a variety of cell types. Further, it is a fundamental part of the anti-viral immune response in the CNS, as shown by IFNα/β-deficient mice having increased neurovirulence and mortality [22]–[24]. Importantly for our work, it has long been known that human cells are non-responsive to murine IFNα/β [25]–[27]; thus, any murine IFNα/β produced by the stroma in conventional MG xenografts in response to infection or injury would have no effect on human xenografts expressing the human IFNα/β receptor. Here we confirmed that human glioma cell lines and mouse glioma cell lines are unresponsive to heterospecific IFNβ, and show that NPcis mouse glioma cell lines are exquisitely sensitive to mouse IFNβ. However, we found that Type-I IFN signalling is not involved in mediating resistance of implanted syngeneic MG to the intracranial injection of MYXV. Rather, our data show that NPcis-derived MGs develop an antiviral state, independent of glioma IFNα/β signalling, that rapidly prevents the replication and spread of MYXV. Our results thus suggest that IFNα/β signalling may be an inappropriate target to overcome MYXV resistance clinically for MG virotherapy. Identifying and overcoming this unique antiviral treatment resistance in the brain will be an important step forward in the preclinical development of MYXV for MG and possible other brain cancers.

## Materials and Methods

### Cell lines and Viruses

NPcis cell lines were derived from C57Bl/6J *Trp53* ^+/−^/*Nf1* ^+/−^ mice [14], [15], [17]. These lines were cultured in Dulbecco's modified Eagle medium (#11965, Invitrogen) containing 10% fetal bovine serum (FBS; Invitrogen) and tested for mycoplasma at regular intervals.

Viruses (vMyx-GFP and vMyx-FLuc) [8]–[10], [12], [28] were propagated and titrated on BGMK cells [29]. UV-inactivated MYXV (dead virus; DV) was prepared by irradiating virus with UV light for 2 hours, and tested for infection of permissive BGMK cells. VSVΔ51 was a kind gift from Dr. John Bell (OHI).

### In vitro infection, viability and titers

To detect viability of NPcis cell lines following MYXV infection *in vitro*, 2×10^3^ cells were plated in 96-well plates overnight, then infected at different multiplicity of infection (**MOI**)s of vMyx-GFP. Cell viability was measured at 48 hours post-infection (**hpi**) by Alamar Blue (Invitrogen) as described by the manufacturer. In experiments using exogenously applied IFNα/β, NPcis lines were pre-incubated in indicated concentration of mouse or human IFNβ (PBL Interferon Source) 6 hours prior to MYXV infection.

Viral replication was qualitatively assessed via early viral gene expression (vMyx-GFP) [8]–[10], [29] at 48 hpi. Cells and GFP fluorescence were observed by microscopy (Zeiss microscope with AxioVision v4.5 Software). Additionally, viral titers were performed by seeding 2.5×10^4^ NPcis or U87 cells in 24-well plates overnight and then treated with MYXV (MOI = 1). The cells and culture media were then collected, freeze-thawed 3 times, and MYXV titered on BGMK cells as previously described [8]–[10], [29].

### Type-I Interferon Measurements

Quantitating functional type-I interferon (IFNα/β) *in vitro* was performed in 96-well plates. NPcis lines were plated overnight, treated with listed stimulus (LMW PolyI∶C from CalTech) and supernatant collected and measured on B16-Blue IFNα/β cells (InvivoGen) following the manufacturers' protocol. To confirm these results at the transcriptional level, RNA was collected (Qiagen RNeasy Plus) from K1492 or K1861, plated in 6-well plates overnight and treated with listed stimulus. cDNA was created with SuperScript II Reverse Transcriptase (Invitrogen) and PCR performed using primers listed in **Table S1**.

To measure IFNα/β production *in vivo* following listed treatments, mice were sacrificed at designated time points and brains removed. The tumour-bearing hemisphere was dissected out, and placed in 500 µL cold PBS in a 1.5 mL microfuge tube. Brains were crushed in the tube using a micropestle, and then sonicated (2×2 sec, 10% Amplitude; Fisher Scientific Sonic Dismembrator Model 500), and spun at 3000× G for 15 minutes. Supernatants were then diluted to normalize protein levels (Bradord Assay; BioRad) and applied to Mouse Verikine Interferon α (#42120) and β (#42400) Elisa kit from PBL Interferon Source.

### IRF9 Knockdown

shRNA constructs in K1492 were created using Sigma Mission IRF9 shRNA (5′- CCGGGTGATGTTTCTCCTTACAAATCTCGAGATTTGTAAGGAGAAACATCACTTTTTG-3′; #TRCN0000232237) or Scrambled (#SHC002) in pLKO.1-puro vector. Briefly, the shRNA vector was co-transfected into HEK-293 cells with pMD2.G (VSV.G env) and pCMV-deltaR8.91. Supernatant was than concentrated ∼50× using Amicon Ultra 100K centrifugal filters (Millipore, Billerica, MA) and added to K1492 cells with 1.6 µg/mL of polybrene overnight. Media was removed and replaced with DMEM (#11965, Invitrogen) with 1.0 µg/mL of puromycin (Invitrogen). Single colony clones of IRF9 knockdown and the Scrambled control were screened for responsiveness to IFNβ after lenti-viral shRNA transduction.

### H&E and Immunohistochemistry

K1492 and K1861 glioma-bearing mice were sacrificed, and brains placed in 10% neutral-buffered formalin for a minimum of 48 hours. Brains were then cut coronally approximately 1 mm behind the injection site and mounted in paraffin blocks, and the first and last slides cut were stained using standard H&E. Immunohistochemistry was performed using the IRF9 antibody (Proteintech, Cat #14167-1-AP; 1∶500) and MT-7e (McFadden Laboratory; 1∶1000) and detected using a biotinylated goat anti-rabbit (Vector; 1∶300) and vectastain elite ABC reagent (Vector, PK-6100). GFAP (Chemicon #MAB360; 1∶500) and S100b (Abcam #ab14849; 1∶100) were detected using the Vector MOM kit (BMK-2202). Slides were mounted, counterstained and viewed with a Zeiss inverted microscope (Axiovert 200M) and a Carl Zeiss camera (AxioCam MRc).

### Orthotopic tumour implantation and intracranial MYXV treatment

Six to eight week-old female wild-type C57Bl/6J mice from Jackson Laboratories (#000664) or CB17 SCID from Charles River were used in this study. The animals were housed in a vivarium maintained on a 12-hour light/dark schedule with a temperature of 22+/−1°C and a relative humidity of 50+/−5%. Food and water were available *ad libitum*. All protocols were reviewed and approved by the Animal Care Committee of the University of Calgary. All animal work procedures were in accordance with the Guide to the Care and Use of Experimental Animals published by the Canadian Council on Animal Care and the Guide for the Care and Use of Laboratory Animals issued by NIH.

For orthotopic injections of K1492 and K1861, the cells were prepared in a 2.5×10^4^ cell/µL PBS solution and 2 µL (5×10^4^) cells implanted into the right striatum of mice as described previously [8], [10], [30], [31]. Briefly, mice were anesthetized with ketamine/xylazine and a 0.5-mm burr hole was made 1.5–2 mm right of the midline and 0.5–1 mm posterior to the coronal suture through a scalp incision. Stereotactic injection used a 10 µL syringe (Hamilton Co., Reno, NV) with a 30-gauge needle, inserted through the burr hole to 3 mm, mounted on a Kopf stereotactic apparatus (Kopf Instruments, Tujanga, CA). The needle was left in place for 60 seconds, and then slowly withdrawn over a 60 second period. For survival studies, animals were followed until they lost 20% of body weight or had trouble ambulating, feeding, or grooming.

Intracranial injection of 5×10^6^ FFU of vMyx-FLuc or vMyx-GFP was performed on day 14 for the K1492 and K1861 lines, unless otherwise stated. Virus was intratumourally administered using the same stereotactic technique as described above, and through the same burr hole created for the tumour implantation.

### In vivo viral replication and bioluminescence imaging

Viral recovery from the tumour-bearing mice was done at day 0 (1 hours post-infection), 1, 3, 5, and 7 days post-infection. Animals were sacrificed and the tumour-bearing hemisphere crushed in the 500 uL of cold PBS using a small pestle, and then sonicated (2×2 sec, 10% Amplitude; Fisher Scientific Sonic Dismembrator Model 500), and spun at 3000× G for 15 minutes and frozen. Supernatants were thawed and then titred on BGMK cells as described above.

Bioluminescence from the vMyx-FLuc-infected cells was imaged with the Xenogen IVIS 200 system. Data were analyzed by drawing a region of interest around the entire skull and measuring the total luminescent emission from that area.

### Statistical Analysis

All data were processed and graphed in either MS Excel 2010 or Prism GraphPad v5.0. Statistics were also performed in these programs. All T-tests were two-sided and values were considered to be statistically significant at p<0.05. Survival curves were generated by the Kaplan-Meier method, and stats were determined using the Log-rank Mantel-Cox test for all treatments together unless otherwise noted.

## Results

### NPcis cell line infection with MYXV in vitro

**Figure 1:**
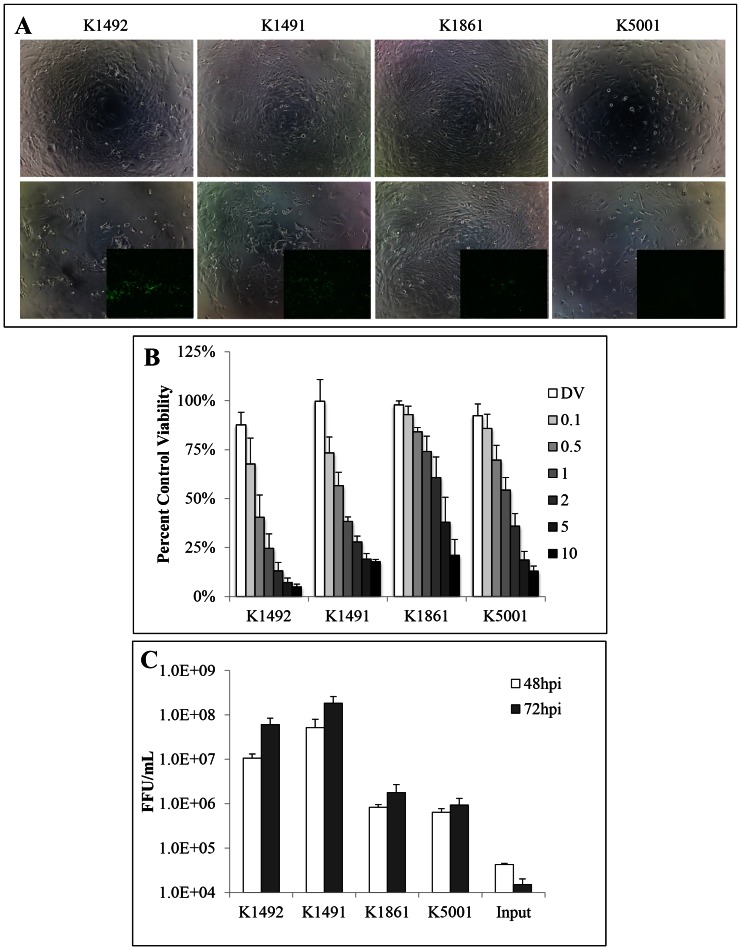
NPcis cell lines have variable susceptibility to MYXV infection and MYXV-mediated cell death in vitro. **A** – NPcis cell lines infected with 1.0 MOI vMyx-GFP (bottom row; 100× phase/contrast, 25× GFP inlay) or control (top row; 100× phase/contrast) at 48 hrs post-infection. **B** – Percent viability of NPcis cell lines at 48 hpi with vMyx-GFP as measured by Alamar blue. **C** – Viral recovery titred on BGMK cells from infected NPcis cell lines at 48 and 72 hpi. Input virus is vMyx-GFP seeded in wells with no cells. Error bars represent standard error and n = 4 for both experiments.

NPcis cell lines K1491, K1492, K1861 and K5001 were assayed for cell viability and viral replication after MYXV treatment *in vitro*. Despite these tumours being derived from the same genetic background and having the same TP53 and NF1 null driving mutations [14], [15], [17], we found a large variability in MYXV infection and replication (**Figure 1**). K1492 and K1492 provided the most robust viral replication, producing high titres of 6.1×10^7^ and 1.8×10^8^ at 72 hour-post-infection (**hpi**). These lines were also the most sensitive to viral treatment, with 1 MOI resulting in 25% and 38% viability at 48 hpi, respectively. Conversely, K1861 was the most resistant to cell death with 74% viability at 1 MOI at 48 hpi, but was killed by the virus at higher MOIs (21% viability with 10MOI, 48 hpi). K5001 had intermediate sensitivity to viral mediated cell death. These more resistant cell lines markedly differed in their ability to replicate the virus, with K1861 and K5001 unable to reach the high titres seen in the other lines, but still able to produce functional virions following infection.

### NPcis lines orthotopically implanted in C57Bl/6 mice and treated with Myxoma virus

**Figure 2:**
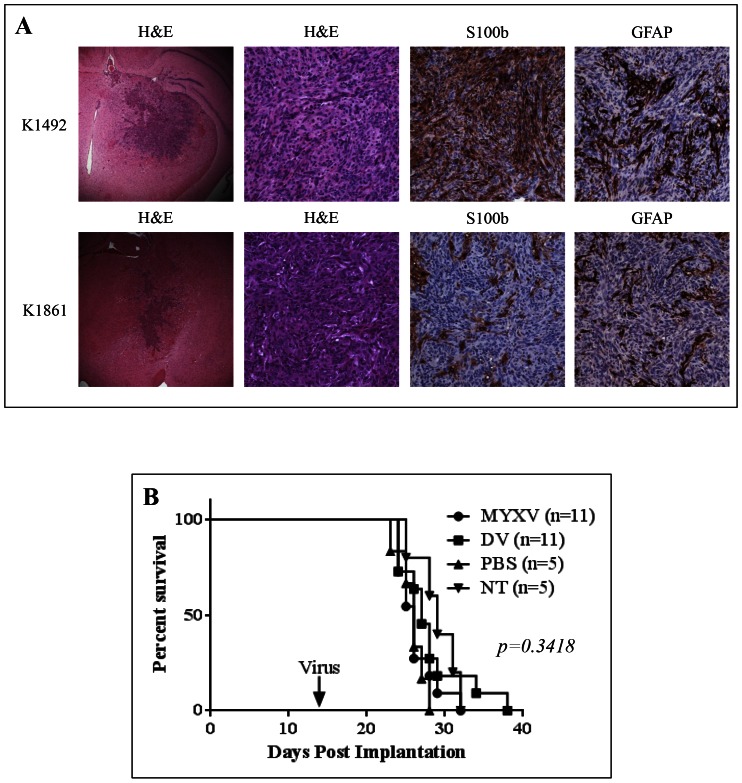
K1492 and K1861 form aggressive intracranial tumours and MYXV treatment results in no efficacy and minimal viral infection with no viral replication. **A** – Histopathology of 14 day K1492 and K1861 by H&E (first column 25×, second column 200×) and astrocytic markers S100b (200×) and GFAP (200×). 5×10^4^ cells of K1492 (**B**) and K1861 (**C**) were intracranially implanted in C57Bl/6J mice and received 5×10^6^ PFUs vMyx-FLuc (MYXV), UV-inactivated virus (DV), PBS, or no treatment (NT) on day 14. **D** – Luciferase measured (Total FLUX) from region-of-interest around the entire mouse skull following 5×10^6^ PFUs vMyx-FLuc in K1492 (n = 8), K1861 (n = 10) or no tumour (n = 5). Error bars represent standard error. **E** – Viral recovery from K1492 (n = 4) and K1861 (n = 4) tumours following intracranial treatment with vMyx-FLuc. Input virus represents mice where virus was recovered 1 hour post-injection. Error bars represent standard error. **F** – Immunohistochemical staining for early MYXV protein MT-7e in 14 day K1492 at 1, 3 and 7 days post-treatment (Top row 25×; Bottom row 100×).

To establish which lines we would utilize for *in vivo* studies, we implanted 5.0×10^4^ cells in the right striatum of C57Bl/6J mice. All mice succumbed to tumour burden, but varied in length of survival (**Figure S1**). Only K1492 and K1861 reproducibly produced intracranial tumours, whilst K1491 and K5001 both tended to produce large extracranial tumour masses with or without a corresponding intracranial tumour. K1492 and K1861 orthotopically implanted in C57Bl/6J mice produced aggressive tumours (**Figure 2A**). Despite having protruding tendrils into the adjacent brain parenchyma, these tumours appear to have a defined tumour border and overall lacked the single cell infiltrate pattern that can appear in astrocytic tumours. Microscopically, these tumours were composed of spindle cells arranged in bundles with a focal storiform pattern. Immunohistochemistry shows patchy expression of GFAP and S100b protein in tumour cells (**Figure 2A**), a feature seen in a some MG patients [32]. The histologic features and immunohistochemical profile are consistent with a gliosarcoma, a glioblastoma variant that has a similar evolution and prognosis. It is easy to speculate that this morphological change to a more mesenchymal phenotype was acquired through culture of the primary cells in serum [33]–[35], since it has been shown that the sarcomatous component in glioblastoma represents an aberrant differentiation of the glioblastoma cells [32]. As the main purpose of this study was to identify intracranial glioma models in immunocompetent C57Bl/6J mice for the pre-clinical modelling of OVs, we deemed K1492 and K1861 relevant for use in our subsequent experiments.

K1492 and K1861 were treated with an intratumoral injection of 5×10^6^ PFU of an MYXV construct that expresses Firefly luciferase, vMyx-FLuc, at 14 days post-implantation (**Figure 2B, C**), a dose we have shown previously to result in efficacy in other intracranial models [8], [9], [28]. These figures represent two separate experiments of K1492 and K1861 amalgamated into one figure. All mice succumbed to tumour burden with no therapeutic benefit [26, 27, 26, and 29 days median survival for MYXV, dead virus (**DV**), PBS or No treatment (**NT**), respectively, for K1492 (Log-rank Mantel-Cox test; p = 0.3418), and 43, 44, 41, and 45 days median survival in K1861 (Log-rank Mantel-Cox test; p = 0.6653)]. Further, two treatments of 1×10^7^ PFU of vMyx-FLuc at 7 and 14 days in K1492, the most susceptible line *in vitro*, showed no therapeutic benefit (26.5 and 28.5 median survival for DV and MYXV, respectively, p = 0.1198; **Figure S2A**). There was no viral replication as measured by viral luciferase activity (**Figure 2D**), with complete clearance of bioluminescent activity by 5 days post-treatment. These viral clearance kinetics closely mimicked viral recovery assays from the tumour (**Figure 2E** **)**. Interestingly, the viral FLUX measured in these models did not differ from non-tumour-bearing animals (**Figure 2D**), and seemed to be a consequence of minor off-target infection of cells in the ventricles (**Figure S2B**) as previously described with intraventricular MYXV administration [36]. However, IHC staining for the early MYXV viral protein, M-T7, found that there was indeed a small proportion of the tumour infected at 1 day-post treatment, with very little evidence of viral protein at 7 days-post treatment (**Figure 2F**). These results demonstrate that the input virus underwent only very transient infection of these tumours, without any sustained viral replication in the tumour beds, and no measurable therapeutic benefit.

### The role of Type-I Interferon in mediating MYXV treatment resistance

**Figure 3:**
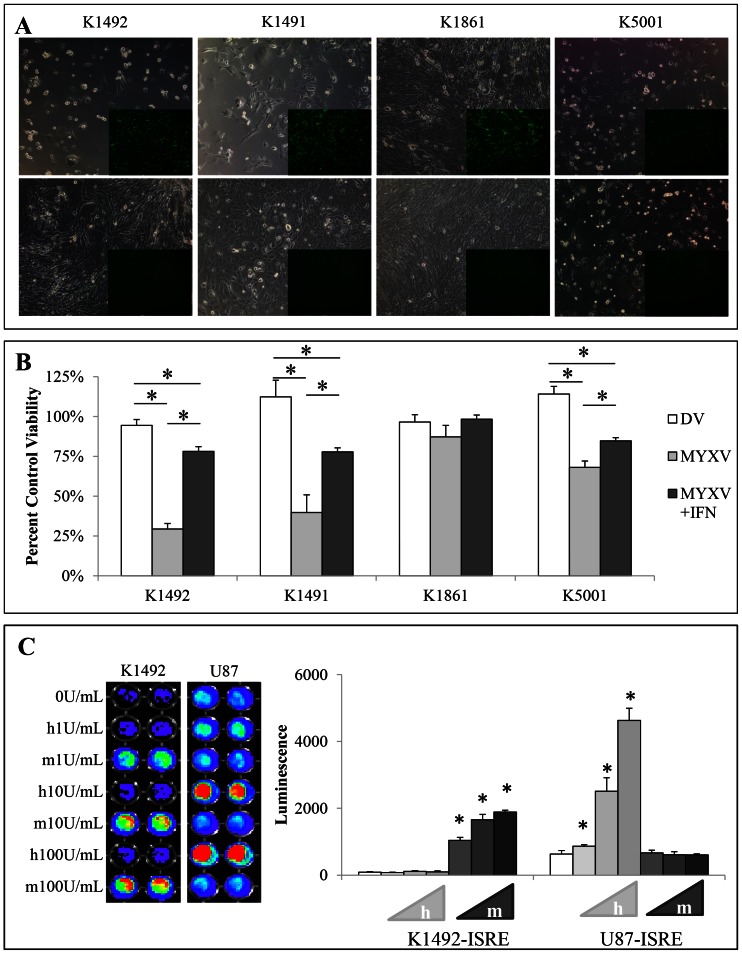
NPcis cell lines are protected by exogenous mouse IFNβ and mouse IFNβ is non-functional on human glioma cell lines. **A** – NPcis cell lines infected with 1.0 MOI vMyx-GFP (top row; 100× phase/contrast, 25× GFP inlay) or 1.0 MOI MYXV-GFP pretreated with 1.0 units of mouse IFNβ (bottom row; 100× phase/contrast, 25× GFP inlay) at 48 hpi. **B** – Percent viability corresponding to controls (MYXV and DV to no treatment control; MYXV+IFN to IFN alone control) of NPcis cell lines at 48 hpi with 1.0 MOI vMyx-GFP or 1.0 MOI vMyx-GFP pretreated with 1.0 units mouse IFNβ as measured by Alamar blue. Error bars represent standard error and asterisks p<0.05 compared to control. **C** – Mouse or human IFNβ on K1492 or U87 lines which stably express an ISRE::FLUC construct. Graph is quantified from representative picture (left). Error bars represent standard deviation and asterisks p<0.05 between groups indicated by the line.

We have previously shown that we can cure or significantly extended lifespan in CB17 SCID or Nude mice bearing human xenografts of conventional [8] or primary neurosphere cell cultures [28]. A preliminary experiment implanting K1492 into CB17 SCID mice showed no treatment efficacy (**Figure S2C**), suggesting to us that species-specific innate immune signalling could be involved in this mechanism. Type-I interferon (IFNα/β) is the quintessential antiviral cytokine [22]–[24], and we hypothesized that the production of IFNα/β, particularly by the tumour stroma, was responsible for *in vivo* treatment resistance. We first determined if the NPcis cell lines were protected by mIFNβ from MYXV viral infection *in vitro*, and found protection with as little as 1 U/mL of mIFNβ (**Figure 3A, B**). This demonstrated that mIFNα/β production from the tumour or stroma in syngeneic models had the potential to protect the tumour from viral infection. We confirmed that murine IFNα/β does not interact with the human IFNα/β receptors [25]–[27] in gliomas using the human U87 cell line and murine K1492 cell line (**Figure 3C** **, S3A**). This suggested to us that any IFNα/β made by the murine stroma in response to MYXV could protect the murine but not human glioma grafts, and explain the treatment discrepancy between xenografts and syngeneic models.

**Figure 4:**
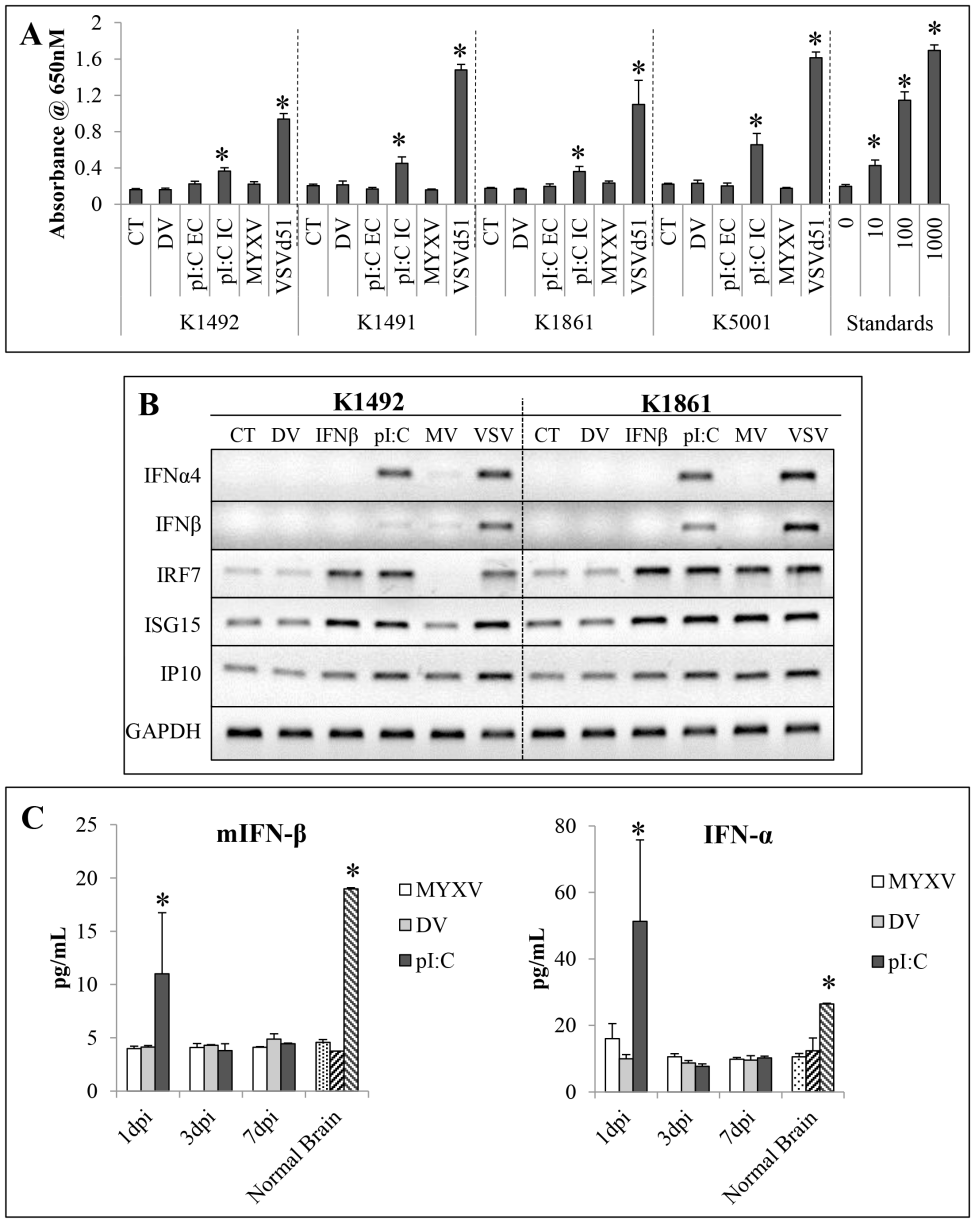
NPcis cell lines do not produce any functional Type-I IFN in vitro nor does MYXV treatment produce a Type-I IFN response in vivo. **A** – Type-I interferon production in NPcis cell lines in response to 1.0MOI MYXV-GFP (MYXV), 1.0 MOI of oncolytic vesicular virus (VSV-Δ51), 25 ug/uL delivered extracellulary (pI∶C EC), 1.0 µg/uL polyI∶C delivered by intracellularly via lipofectimine (pI∶C IV), UV-inactivated MYXV (DV) or no treatment (CT) as measured using the HEK-Blue system 24 hpi. Error bars represent standard error and asterisks p<0.05 compared to control. **B** – Reverse-transcriptase (RT)-PCR of Type-I interferons and Type-I interferon responsive genes in K1492 and K1861 lines in response to 1.0 MOI vMyx-GFP (MYXV), 1.0 MOI of oncolytic vesicular virus (VSV), 1.0 µg/uL intracellular polyI∶C (pI∶C), 10 Units of exogenous mouse IFNβ (IFNβ), UV-inactivated MYXV (DV) or no treatment (CT). **C** - Elisa of mouse IFNβ and mouse pan IFNα from 14 day K1492 tumours 1, 3 or 7 days post intracranial administration of 5×10^6^ PFU vMyx-FLuc (MYXV), UV-inactivated MYXV (DV) or 5 µg of naked polyI∶C (pI∶C), n = 2 per time point. Stippled bar represents K1492 tumour at 14, 15, 17, and 21 days with combined with no treatment (n = 2 per time point). Left-slashed bars are normal mouse brain (n = 2) and right-slashed bars are normal mouse brain spiked with 15 pg and 20 pg of IFNβ or IFNα, respectively. Error bars represent standard error and asterisks p<0.05 compared to normal brain.

We then determined if IFNα/β was produced in the NPcis lines *in vitro* by MYXV infection. However, MYXV infection did not induce the production of any functional IFNα/β in any of the lines (**Figure 4A**). This is similar to our previous results with all the human cell lines we have tested, including primary glioma neurosphere and primary human fetal astrocyte cultures, which fail to show any detectable IFNα/β production, despite, for the most part, being able to make IFNα/β in response to other stimuli [28]. To confirm these results we performed reverse-transcriptase PCR on lines K1492 and K1861 (**Figure 4B**), and found that after 24-hours of exposure to MYXV both lines had no significant transcriptional activation of the IFNβ and IFNα4 genes, while both VSVΔ51 or polyI∶C, both strong activators of IFNα/β, showed robust IFN responses. The time point of 24 hpTx also allowed us to look at auto/paracrine IFNα/β signalling, and showed that only K1861 had transcriptional activation of interferon-stimulated genes IRF7, ISG15 and IP10. The activation of these genes in the absence of functional IFN production suggests that perhaps these are activated upon infection independent of type-I IFN production [37], [38]. Interestingly, this only occurred in the resistant K1861 cell line and, thus, could be part of the mechanism mediating this *in vitro* resistance.

Given the lack of IFNα/β produced by the lines in response to MYXV infection *in vitro*, we next wanted to determine if stromal IFNα/β production might have occurred *in vivo*. We measured IFNα/β production *in vivo* by ELISA in MYXV-treated K1492-bearing C57Bl/6 mice. We were surprised to find no significant increase in IFNα/β following treatment (**Figure 4C**); this is particularly striking in comparison to the response to a small amount of naked polyI∶C (5ug) administered intracranially. Together with our *in vitro* data, this suggests that virus-induced IFNα/β is not involved in the *in vivo* resistance mechanism.

**Figure 5:**
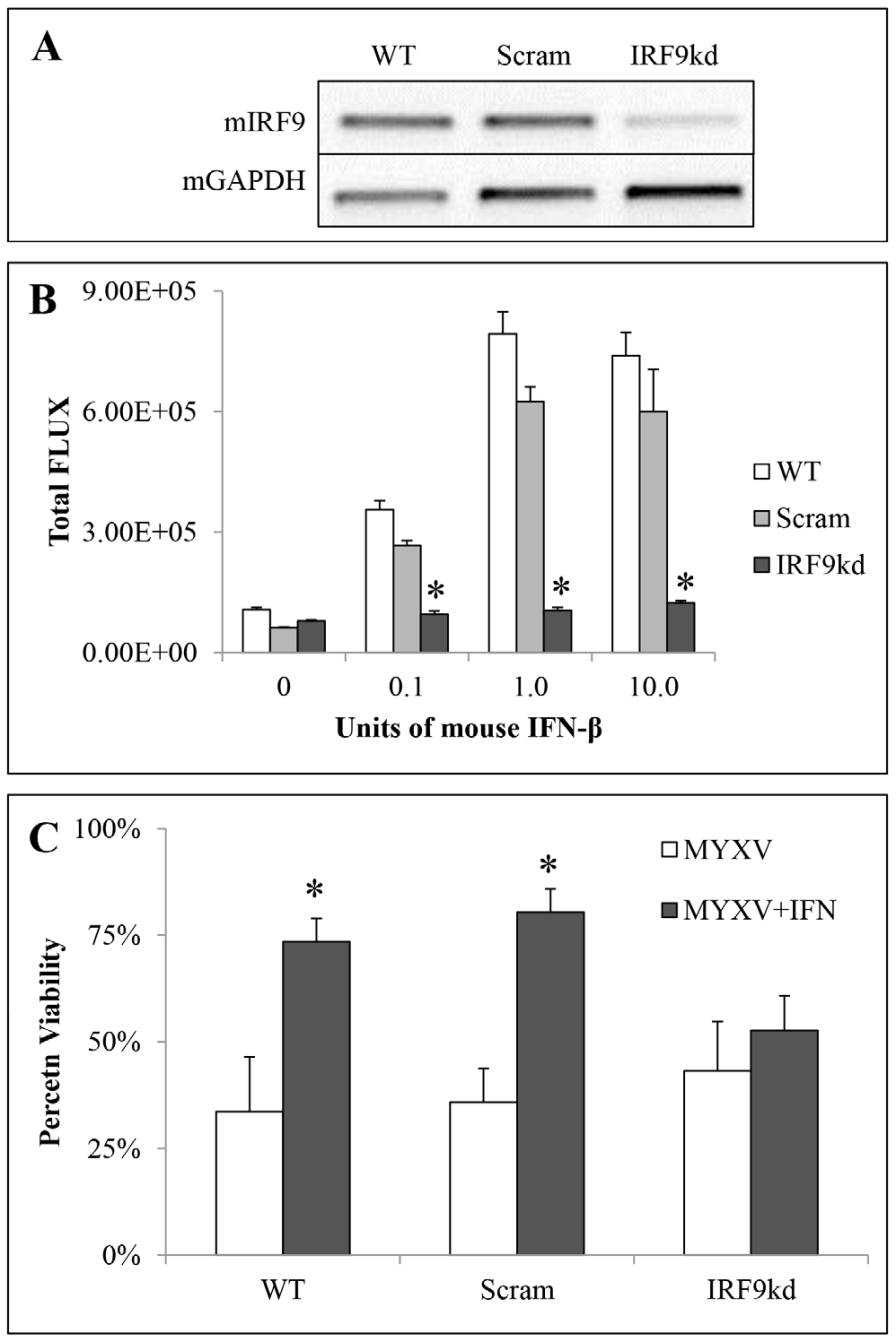
IRF9 knockdown results in loss of protection of IFNβ on K1492 in vitro. **A** – RT-PCR of IRF9 message level after stable transduction of IRF9 shRNA (IRF9kd), scrambled control (Scram) or wildtype (WT) K1492. **B** – Luminescence activity of an inducible luciferase construct stably transfected into IRF9 knockdown (IRF9kd), scrambled control (Scram) or wildtype cells (WT) K1492 cells. Error bars represent standard error and asterisks p<0.05 when compared to WT K1492. **C** – K1492 knockdown construct or controls 48 hpi with 1.0 MOI vMyx-GFP with or without the pretreated with 1.0 units of mouse IFNβ as measured by Alamar blue. Error bars represent standard error and asterisks p<0.05 when compared MYXV alone.

To show definitively that IFNα/β production by the tumour or the stroma was not involved in mediating the *in vivo* resistance to MYXV, we reduced K1492's ability to respond to IFNα/β by silencing IRF9 with a shRNA construct transduced via lentiviral infection. Knockdown of IRF9, as a central mediator of IFN signalling, would also attenuate any anti-viral crosstalk from Type-II and III IFN produced by the tumour or stroma, which could also possibly mediate this resistance. We successfully knocked-down IRF9 in K1492 (**Figure 5A**), which resulted in a functional loss of transcriptional activity at interferon-response elements (ISRE) as measured by an ISRE::FLUC reporter (**Figure 5B** **, S3B**). Importantly, this IRF9 knockdown resulted in a dramatic loss of the protection provided by the application of exogenous IFNβ *in vitro* (**Figure 5C** **, S3C**).

**Figure 6:**
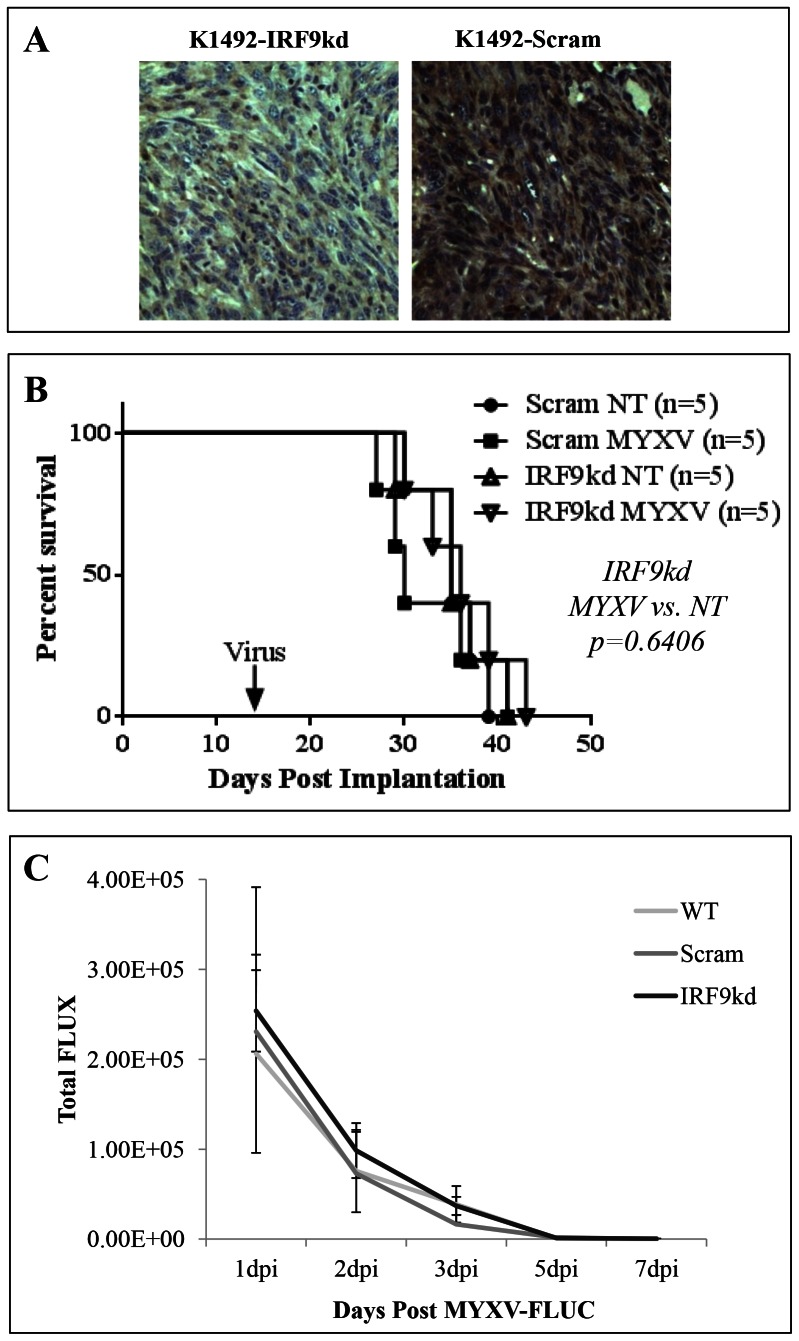
K1492 IRF9 knockdown in vivo results in no change in efficacy or viral replication. **A** – Immunohistochemical confirmation of IRF9 knockdown in K1492 tumours 14 days post-implantation (200×). **B** – Survival of 5×10^4^ cells of K1492 IRF9 knockdown (IRF9kd) or Scrambled control (Scram) in C57Bl/6J mice receiving 5×10^6^ PFUs vMyx-FLuc (MYXV) or no treatment (NT) on day 14. **C** – Luciferase measured (Total FLUX) from region-of-interest around the entire mouse skull following 5×10^6^ PFUs vMyx-FLuc in K1492 control (WT), scrambled control (Scram) or IRF9 knockdown (IRF9kd) on day 14. n = 5 for each group and error bars represent standard error.

To determine if ablating IFN signalling *in vivo* would overcome resistance to MYXV *in vivo*, we implanted the K1492 IRF9 knockdown or its scrambled control into C57Bl/6J mice. Importantly, we found that the knockdown persisted *in vivo* through immunohistochemistry for IRF9 (**Figure 6A** **, S4**), and that the knockdown had similar tumour growth kinetics to the K1492 scrambled control. Treatment with vMyx-FLuc resulted in no change in survival (35 days for no treatment versus 30 days for MYXV treatment in Scram-K1492 (Log-rank Mantel-Cox test; p = 0.8279) and 35 days for no treatment versus 36 days for MYXV treatment in IRF9kd-K1492 (Log-rank Mantel-Cox test; p = 0.6406); **Figure 6B**), with no change in viral luciferase (**Figure 6C**). These experiments, coupled to the observations of no significant IFNα/β production *in vitro* and *in vivo*, strongly suggest that the *in vivo* resistance to MYXV therapy in the intracranial environment of immunocompetent mice is independent of the quintessential anti-viral IFNα/β signalling.

## Discussion

Our study uses a panel of primary, syngeneic mouse glioma cell lines to determine the mechanisms of glioma resistance to virotherapy with a candidate oncolytic virus in preclinical models. As we have previously shown in syngeneic rodent models [10], MYXV treatment demonstrates neither replication nor survival benefit in these orthotopically grafted murine gliomas *in vivo*, despite being sensitive to MYXV-mediated cell death and viral replication *in vitro*. This is in stark contrast to what we have found in xenograft models with traditional human glioma cell lines [8] and with freshly cultured patient neurospheres [28], where we found robust viral replication and survival benefit from MYXV treatment when these cells are grown in immunodeficient mice. This resistance of syngeneic MG tumours to MYXV is also in contrast to the situation for other classes of cancer localized outside the brain, such as for disseminated pancreatic cancer in the peritoneal cavity, where MYXV virotherapy is most effective against syngeneic murine Pan02 tumours in C57/Bl6 mice, compared to the reduced efficacy against the same cells engrafted into the intraperitoneal cavity of immunodeficient mice [12]. Thus, we have investigated this apparently brain-specific blockade to MYXV replication and anti-tumour responses within the intracranial environment of the immunocompetent host.

The murine glioma lines we used in this manuscript are derived from spontaneous low-high grade gliomas that arose in *Trp53* ^+/−^/*Nf1* ^+/−^ C57Bl/6J mice [14], [15], [17]. As such, these lines fill an underrepresented niche in the experimental glioma therapeutics field that is of special interest to investigators interested in oncolytic viruses, immunotherapies, or in stromal contributions to treatment. In this model, tumourigenesis is initiated by loss-of-heterozygosity at the *Trp53* and *Nf1* loci, leading to the development of low-grade astrocytomas, which can progress to diffusely infiltrative high-grade gliomas [14]. The relevance of the tumour suppressors *Trp53* and *Nf1* to glioma biology has been robustly validated in comprehensive genomic studies. Recent characterization of the mutational profiles of high-grade human gliomas by the Cancer Genome Atlas (TCGA) Research Network demonstrated that 23% of patient samples harboured somatic *NF1* inactivating mutations or deletions, of which 64% represented *NF1* heterozygous deletions [39]. The relevance of *NF1* to human high-grade gliomas was further validated when the TCGA identified *NF1* as the defining mutation for the mesenchymal subtype of glioblastoma in a multi-dimensional genomic analysis [19]. Mutation of the human p53 gene, *TP53*, is the most frequent genetic alteration in precursor low-grade astrocytomas (present in 60% of cases) [40] and was also shown to be frequently mutated in both the mesenchymal subtype and classical subtype of glioblastoma [19]. In addition to *TP53* and *NF1* mutations, prior *in vitro* characterization of the *Trp53* ^+/−^/*Nf1* ^+/−^ C57Bl/6J-derived cell lines revealed concomitant over-expression of commonly amplified receptor tyrosine kinases, including EGFR and PDGFRα [17]. The accumulation of aberrations in gene expression accurately recapitulates the clinical progression from low- to high-grade gliomas in patients, whereby loss of p53 function in low-grade astrocytomas precedes further genetic and gene expression changes that ultimately lead to progression and development of high-grade gliomas [40]. We consider it very important to preclinically test OVs in cell lines that contain relevant genomic alterations, as these changes may ultimately determine the susceptibility to viral infection, viral replication, and viral-mediated cell death.

Secondly, these lines arose in C57Bl/6J mice, and thus are ‘graftable’ into the most common immunocompetent laboratory mouse strain. The power of using this strain of mouse is the numerous transgenic and knockout animals available, allowing for the interrogation and modelling of glioma-stromal interactions that can influence tumour-development and treatment response. Utilizing transgenic mice for the precise and complete ablation of specific aspects of the immune system or other aspects of the glioma microenviroment (ie, ECM components) allows for comprehensive studies that can interrogate exactly how these therapies work and can be improved.

Discovering minimal MYXV infection in the tumour *in situ* with no sustained viral replication, although not surprising, is worrisome if we extrapolate this data for the clinical use of virotherapy of gliomas and other brain cancers. We have recently published a review article summarizing the clinical experience of OVs for glioma treatment [5]. Of the currently completed trials only two [6], [7], both with a neuroattenuated HSV, were designed to investigate viral infection and possible replication in the tumour. These results, with a combined total of 21 patients with their tumours resected or biopsied 2–9 days post-intratumoural virus inoculation, only found recovery of functional virus in three cases. Further, IHC staining for viral proteins was only found in 4 of the 21 patients. Both of these strains of HSV produced robust viral infection or replication and/or measurable efficacy in mouse subcutaneous or orthotopic xenografts [41], [42]. Interestingly, HSV-G207, as well as other unarmed HSV-strains, was found to provide measurable survival benefit in an orthotopic syngeneic B6D2F1 glioma model, albeit only when infecting small tumours early after implantation [21]. Of note in that study was the *in vivo* viral clearance kinetics closely mimicked what occurs in our model, and, ultimately, what was seen in patients. Collectively, this suggests to us that syngeneic models more closely recapitulate the clinical experience with OV therapy for gliomas, and further understanding and optimization of these therapies should be robustly interrogated using these or similar syngeneic systems as we advance these treatments.

There has been much development in the area of tumour-stroma interactions dictating treatment resistance, with much recent interest in how hepatocyte growth factor (HGF) can mediate BRAF-inhibitor resistance in melanoma [43], [44]. Although growth factors are largely thought to be cross-reactive between mouse and human, many chemokines and cytokines have species-specific interactions, such as Type-I [25]–[27] and Type-II [45] interferons and members of the TNFα family [46]. These interactions may be of great importance to therapeutic outcome, especially for immune-based therapies such as OVs, and thus models where these interactions are conserved should become part of the standard for preclinical testing. Of great importance here is the incompatibility of mouse Type-I interferon (IFNα/β) and the human IFNα/β receptor. Some would argue that in many cancer cells, compromised IFNα/β signalling occurs as a consequence of transformation [47], [48]. Indeed, this seems to be the case in some cell lines, but many gliomas, as seen in the NPcis cell lines, seem to retain their ability to both produce and respond to Type-I IFNα/β [10], [28], [49], [50]. This is one of the main reasons for the pursuit of IFNα/β's anti-proliferative and pro-apoptotic effect as a glioma therapeutic in the clinic [51], in addition to its immunomodulatory functions. Indeed, it could be suggested that if MYXV did mount a robust IFNα/β response in the glioma microenvironment, we may see some indirect therapeutic response in these syngeneic models.

The lack of a MYXV-induced IFNα/β response *in vivo* in the mouse glioma microenvironment is an interesting observation, and strongly suggests that an IFNα/β response is not necessary to protect the tumour or the rest of the brain from MYXV infection. It has previously been shown that STAT1-deficient mice on the 129Sv/Ev background rapidly succumb to intracranial injections of MYXV [52], suggesting that IFNα/β signalling is important in protecting against MYXV neurovirulence in this strain of mouse. Perhaps this is a result of inherent differences between the 129Sv/Ev and C57Bl/6J background of mouse, which have shown strain-specific effects in models of HSV viral encephalitis [53], [54] and experimental autoimmune encephalomyelitis [55], [56]. It would be interesting to look at MYXV neurovirulence in C57BL/6J mice deficient in IFNAR1 or IRF9, which would specifically ablate IFNα/β signalling in these animals.

The mechanism of treatment resistance in the syngeneic NPcis glioma cell lines when implanted intracranially into C57Bl/6J mice are of great interest to our laboratory. It has not escaped our notice that these tumours are highly infiltrated with myeloid-derived and lymphoid-derived cell types before and after intratumoural treatment. In our previous study looking at MYXV in syngeneic rat models [10], we demonstrated that the mTOR inhibitor rapamycin administrated prior to MYXV treatment resulted in increased tumour infection, viral replication and an overall better efficacy then either treatment alone. In that study we found that rapamycin was able to inhibit the MYXV-induced infiltration of CD68+ and CD163+ microglia/myeloid-derived cells. We are currently immunophenotying the glioma microenvironment and using knock-out C57Bl/6J mice to determine which, if any, of these immunocytes are necessary for inhibiting viral infection and replication within these tumours. It will be interesting to see if we lose combination effects with immunosuppressants, such as rapamycin, that have been used to enhance oncolytic viral therapy. These types of experiments will allow a thorough understanding of the mechanisms behind these combination effects, and perhaps lead to more targeted combinations. We believe that by identifying the anti-viral effectors and cell types responsible for resistance in these immunocompetent models, we will be able to modify the treatment regime to include chemotherapeutics or genetic alterations to MYXV that will specifically enhance the ability of oncolytic viruses to treat brain tumours in patients. Further, as MYXV moves closer to a clinical evaluation in MG patients, we will have an understanding of how to translate this knowledge back into patients receiving different therapeutic regimens to see if similar challenges will indeed be the case.

Of concern about these models is the ‘mesenchymal’ drift that seems to have occurred in these cells lines, such that the grafted tumours appear more of a gliosarcoma then a truly infiltrative astrocytoma like the tumours from which they originated [14]–[16]. This has been shown to be a phenomenon associated with primary gliomas cultured in serum [33]–[35]. To address this concern for future studies, we are currently deriving cell lines from NPcis astrocytoma-bearing mice cultured under neurosphere conditions, which have been reported to better retain the original tumour's genetics and phenotypic appearance *in vivo* [33]. These cells may prove to be an even more relevant model for the preclinical testing of glioma experimental therapeutics in an immunocompetent setting. Further, it would be interesting to look at other oncolytic viruses in this model to ascertain if similar limitations exist, and if resistances are independent of IFNα/β. Once mechanisms for treatment resistance are discovered, it will be important to determine if these mechanisms are conserved across all OVs used in glioma preclinical studies.

In conclusion, the use of an immunocompetent syngeneic model of orthotopic murine glioma reveals that an intracranial injection of an oncolytic virus with prospects of clinical application, MYXV, is ineffective at reducing glioma growth. Further, this virus blockade appears to be specific for the brain, and the prominent anti-viral cytokine, IFNα/β, is not responsible for the neutralization of viral activity, suggesting that other mechanisms yet to be uncovered must be identified in order for this OV therapy to have better clinical utility.

## Supporting Information

Figure S1 **NPcis cell lines orthotopically implanted in C57Bl/6 mice succumb to tumour burden.** 5×10^4^ cells of K1492, K1861, K1491 and K5001 implanted into the right straitum of C57Bl/6 mice.
(TIF)

Figure S2 **A** - 5×10^4^ K1492 were implanted into the right striatum of C57Bl/6 mice, and treated with 1×10^7^ PFU of vMyx-GFP (MYXV) or UV-inactivated MYXV (DV) at 7 and 14 days-post implantation. P-value represents result of a Log-rank Mantel-Cox test. **B** - K1492 implanted into C57Bl/6 mice and treated with 5×10^6^ PFU of vMyx-GFP (MYXV), and sacrificed at 1 and 7 days post-infection (dpi). Pictures represent two mice at each time point with the GFP channel alone (left) and the GFP+brightfield (right). Arrows indicate tumour location. **C** - 5×10^4^ K1492 were implanted into the right striatum of C57Bl/6 or CB17-SCID mice and treated with 1×10^7^ PFU of vMyx-GFP (MYXV).
(TIF)

Figure S3 **A** - Specificity of mouse and human IFN using the HEK-Blue system (InvivoGen) which uses an interferon specific promoter upstream of SEAP to detect human IFN. This is the human counterpart of the B16-Blue described in the methods. Graph is quantitated picture where error bars represent standard deviation and asterisks p<0.05 between IFN treatment at control. **B** - Reprehensive picture of luminescence from K1492 wildtype (WT), scrambled shRNA (Scram) or IRF9 shRNA (IRF9kd) stably transfected with a ISRE::FLUC construct and treated with listed doses of IFNβ for 8 hours. **C** - Reprehensive pictures from K1492 wildtype (WT), scrambled shRNA (Scram) or IRF9 shRNA (IRF9kd) controls, infected with 1.0 MOI vMyx-GFP (top row; 100× phase/contrast, 25× GFP inlay), or 1.0 MOI MYXV-GFP pretreated with 1.0 units of mouse IFNβ (bottom row; 100× phase/contrast, 25× GFP inlay) at 48 hpi.
(TIFF)

Figure S4 **Immunohistochemical confirmation of IRF9 knockdown in K1492 tumours 14 days post-implantation (200×).** Representative pictures from three individual mice bearing scrambled shRNA (Scram) or IRF9 shRNA (IRF9kd).
(TIF)

Table S1 **RT-PCR primer sequences.**
(TIF)
