# Correction: Resistance to Oncolytic Myxoma Virus Therapy in Nf1^−/−^/Trp53^−/−^ Syngeneic Mouse Glioma Models Is Independent of Anti-Viral Type-I Interferon

**DOI:** 10.1371/journal.pone.0101827

**Published:** 2014-06-26

**Authors:** The PLOS ONE Staff

Figure 2 is incorrect; panels C-F are missing. The figure legend is correct. The authors have provided a corrected version here.

**Figure 2:**
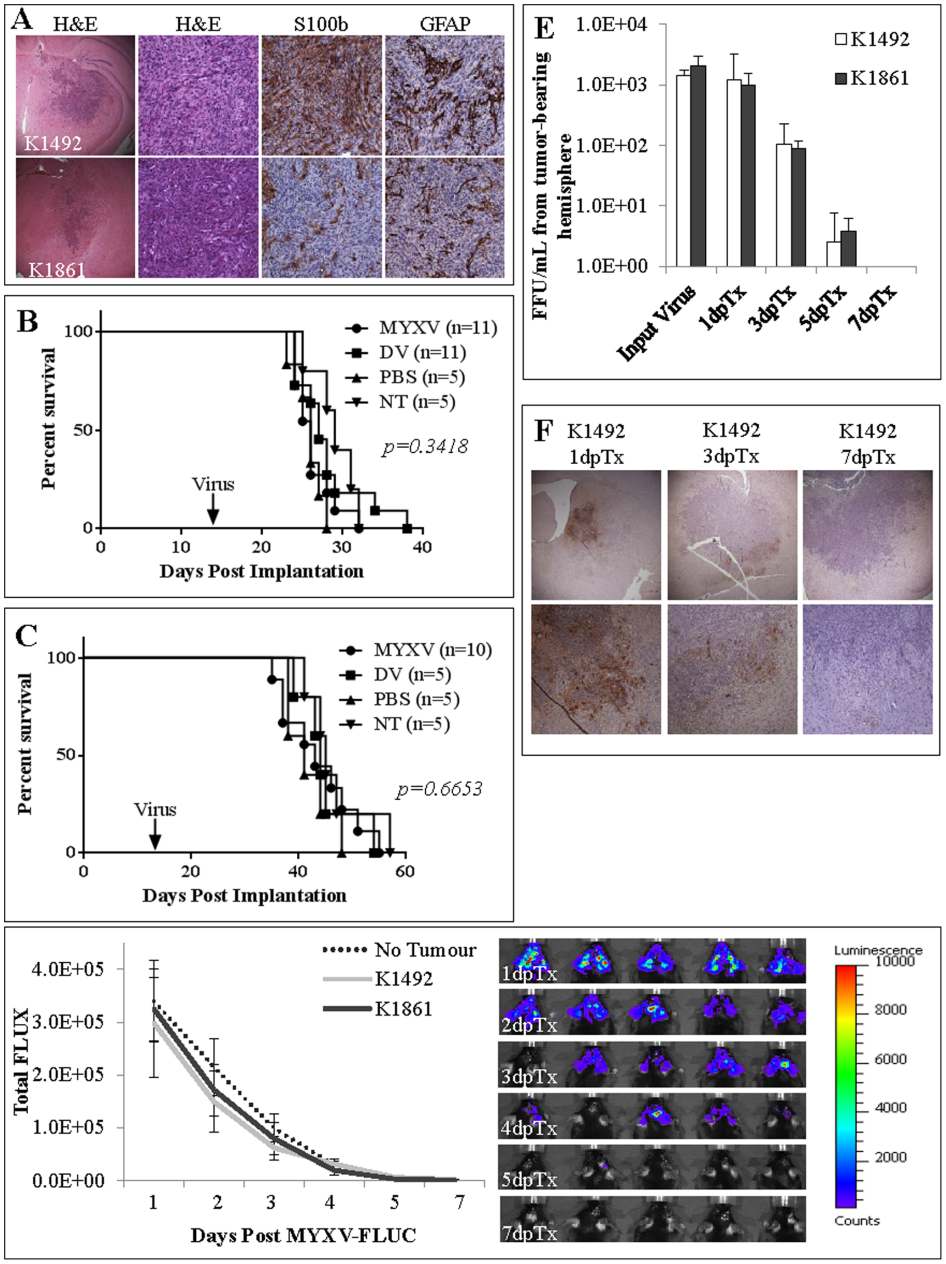
K1492 and K1861 form aggressive intracranial tumours and MYXV treatment results in no efficacy and minimal viral infection with no viral replication. **A** – Histopathology of 14 day K1492 and K1861 by H&E (first column 25×, second column 200×) and astrocytic markers S100b (200×) and GFAP (200×). 5×10^4^ cells of K1492 (**B**) and K1861 (**C**) were intracranially implanted in C57Bl/6J mice and received 5×10^6^ PFUs vMyx-FLuc (MYXV), UV-inactivated virus (DV), PBS, or no treatment (NT) on day 14. **D** – Luciferase measured (Total FLUX) from region-of-interest around the entire mouse skull following 5×10^6^ PFUs vMyx-FLuc in K1492 (n  =  8), K1861 (n  =  10) or no tumour (n  =  5). Error bars represent standard error. **E** – Viral recovery from K1492 (n  =  4) and K1861 (n  =  4) tumours following intracranial treatment with vMyx-FLuc. Input virus represents mice where virus was recovered 1 hour post-injection. Error bars represent standard error. **F** – Immunohistochemical staining for early MYXV protein MT-7e in 14 day K1492 at 1, 3 and 7 days post-treatment (Top row 25×; Bottom row 100×).
