# Supplementary figures and images for: Resistance to Oncolytic Myxoma Virus Therapy in Nf1−/−/Trp53−/− Syngeneic Mouse Glioma Models Is Independent of Anti-Viral Type-I Interferon
Source: PLoS One. 2013 Jun 6;8(6):e65801. doi: 10.1371/journal.pone.0065801 (PMC3675064; doi:10.1371/journal.pone.0065801)

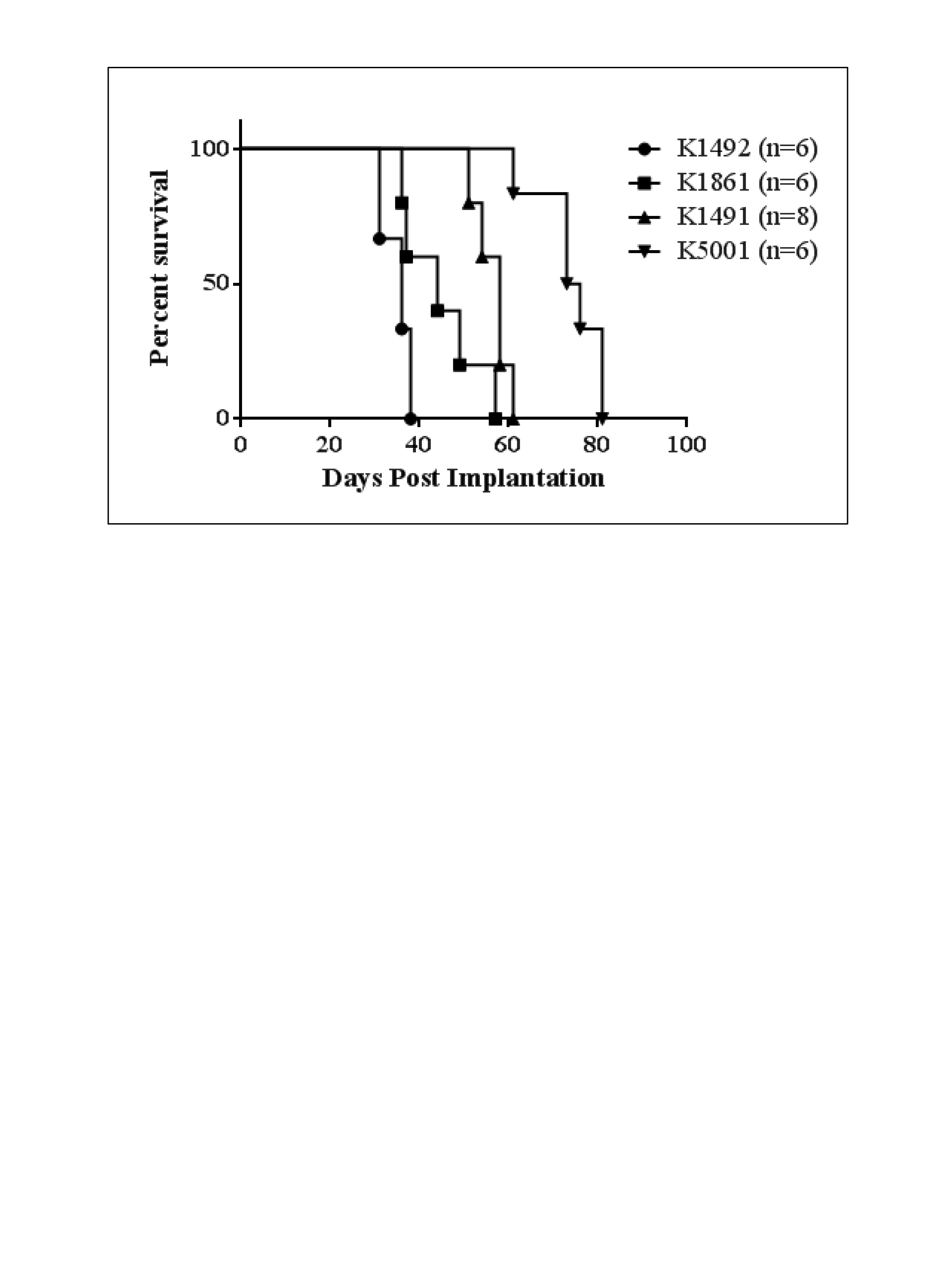

Supplement: Figure S1 — NPcis cell lines orthotopically implanted in C57Bl/6 mice succumb to tumour burden. 5×10^4^ cells of K1492, K1861, K1491 and K5001 implanted into the right straitum of C57Bl/6 mice. (TIF) [file pone.0065801.s001.tif]

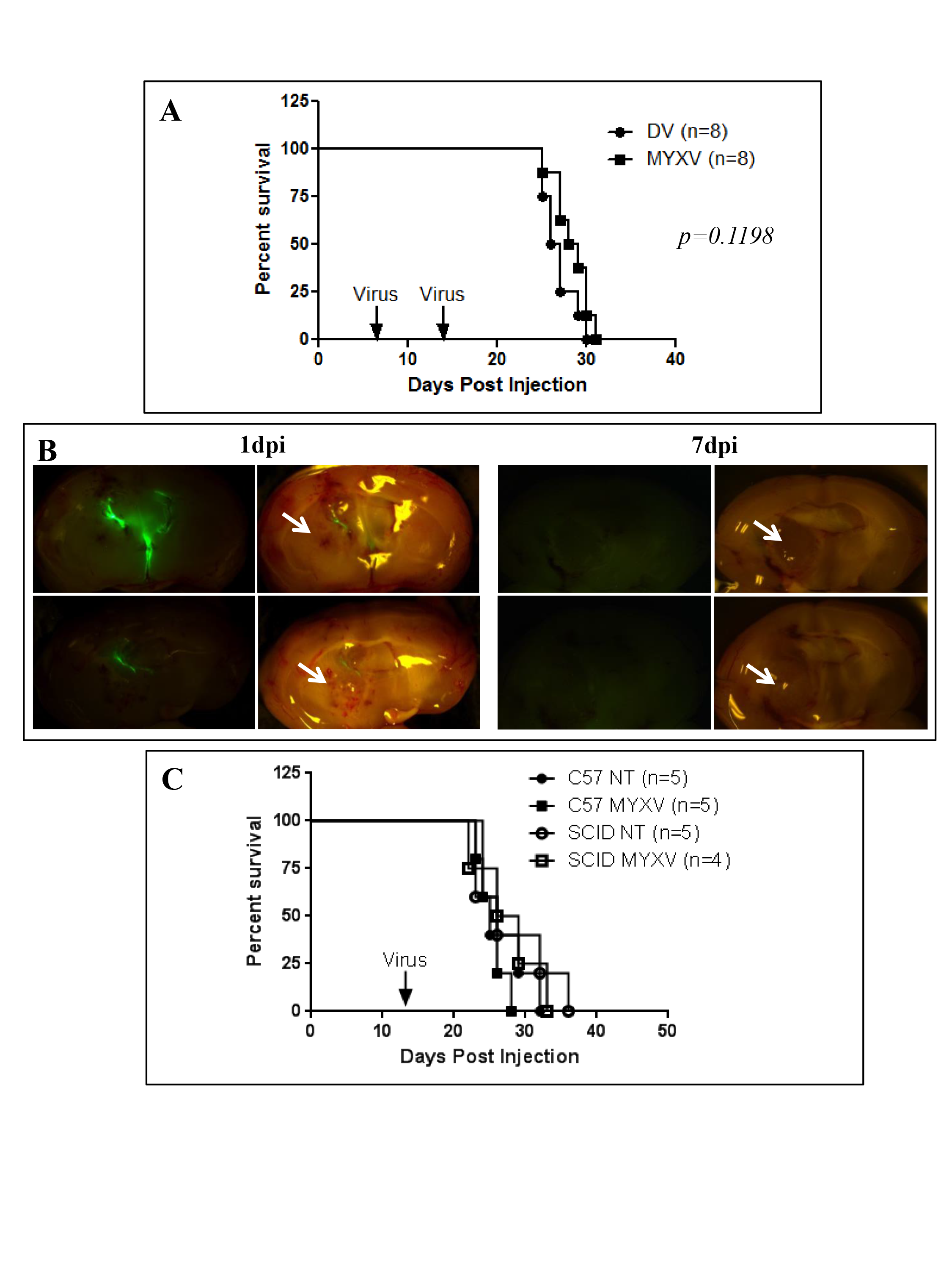

Supplement: Figure S2 — A - 5×10^4^ K1492 were implanted into the right striatum of C57Bl/6 mice, and treated with 1×10^7^ PFU of vMyx-GFP (MYXV) or UV-inactivated MYXV (DV) at 7 and 14 days-post implantation. P-value represents result of a Log-rank Mantel-Cox test. B - K1492 implanted into C57Bl/6 mice and treated with 5×10^6^ PFU of vMyx-GFP (MYXV), and sacrificed at 1 and 7 days post-infection (dpi). Pictures represent two mice at each time point with the GFP channel alone (left) and the GFP+brightfield (right). Arrows indicate tumour location. C - 5×10^4^ K1492 were implanted into the right striatum of C57Bl/6 or CB17-SCID mice and treated with 1×10^7^ PFU of vMyx-GFP (MYXV). (TIF) [file pone.0065801.s002.tif]

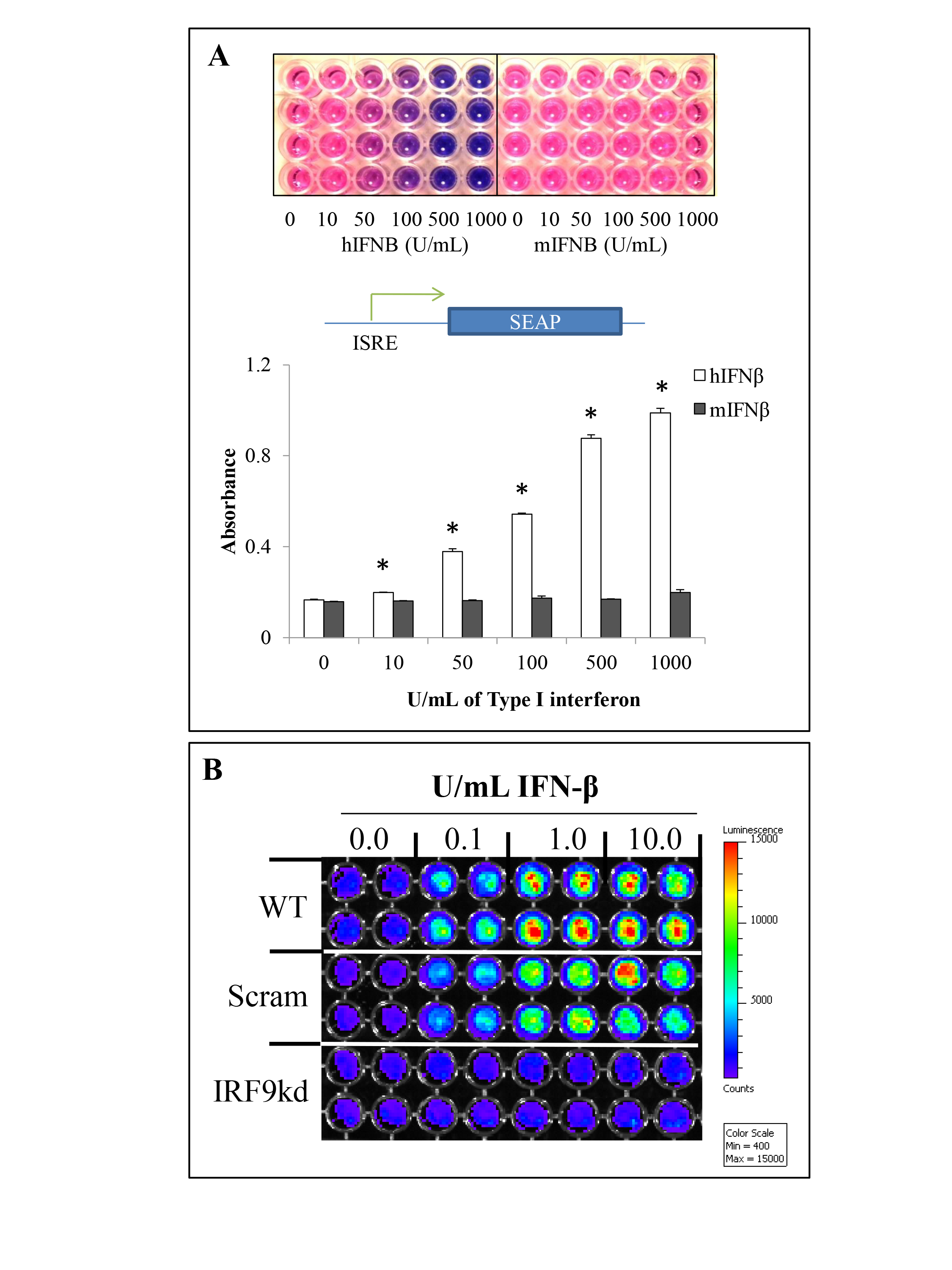

Supplement: Figure S3 — A - Specificity of mouse and human IFN using the HEK-Blue system (InvivoGen) which uses an interferon specific promoter upstream of SEAP to detect human IFN. This is the human counterpart of the B16-Blue described in the methods. Graph is quantitated picture where error bars represent standard deviation and asterisks p<0.05 between IFN treatment at control. B - Reprehensive picture of luminescence from K1492 wildtype (WT), scrambled shRNA (Scram) or IRF9 shRNA (IRF9kd) stably transfected with a ISRE::FLUC construct and treated with listed doses of IFNβ for 8 hours. C - Reprehensive pictures from K1492 wildtype (WT), scrambled shRNA (Scram) or IRF9 shRNA (IRF9kd) controls, infected with 1.0 MOI vMyx-GFP (top row; 100× phase/contrast, 25× GFP inlay), or 1.0 MOI MYXV-GFP pretreated with 1.0 units of mouse IFNβ (bottom row; 100× phase/contrast, 25× GFP inlay) at 48 hpi. (TIFF) [file pone.0065801.s003.tiff]

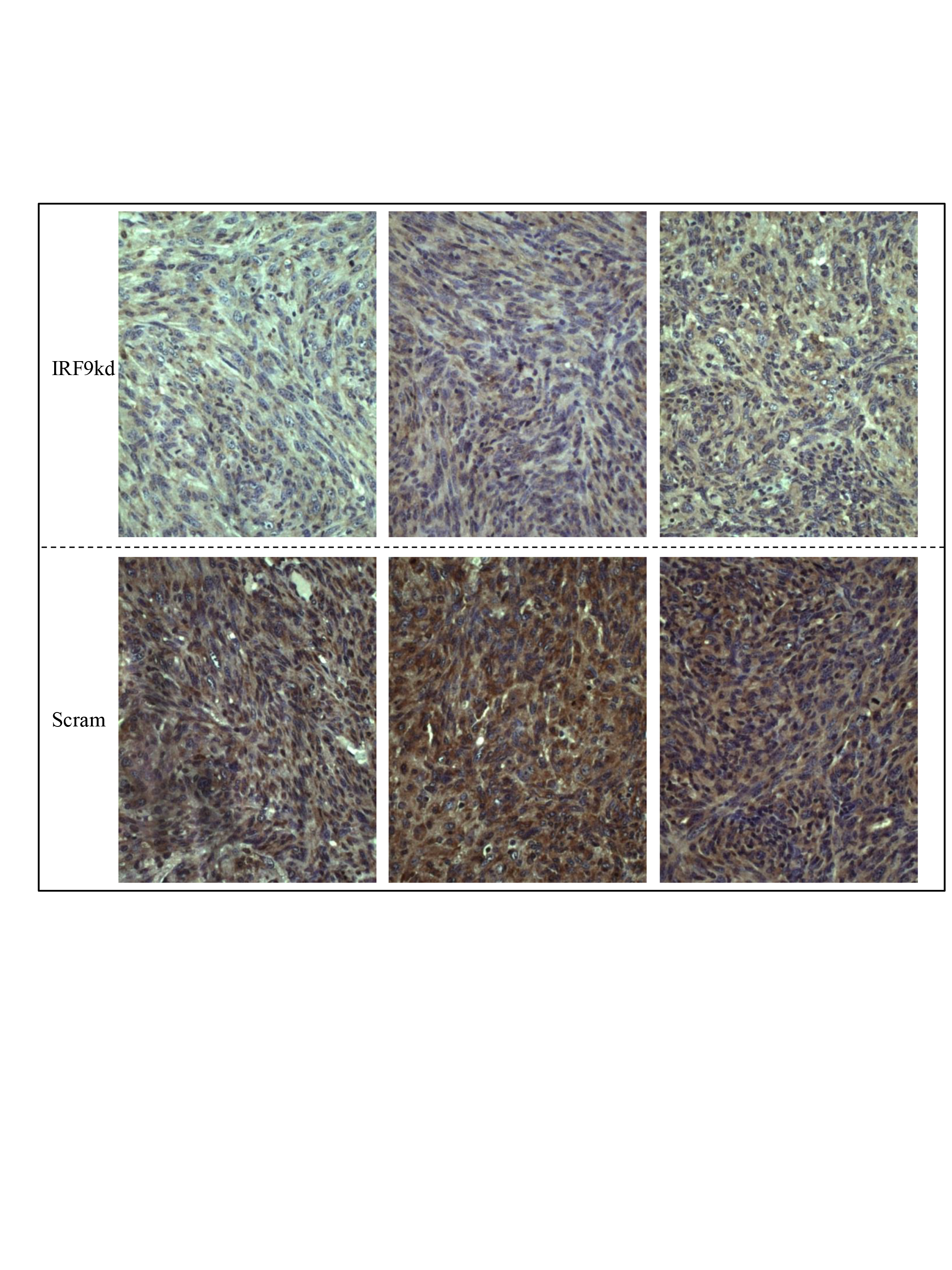

Supplement: Figure S4 — Immunohistochemical confirmation of IRF9 knockdown in K1492 tumours 14 days post-implantation (200×). Representative pictures from three individual mice bearing scrambled shRNA (Scram) or IRF9 shRNA (IRF9kd). (TIF) [file pone.0065801.s004.tif]

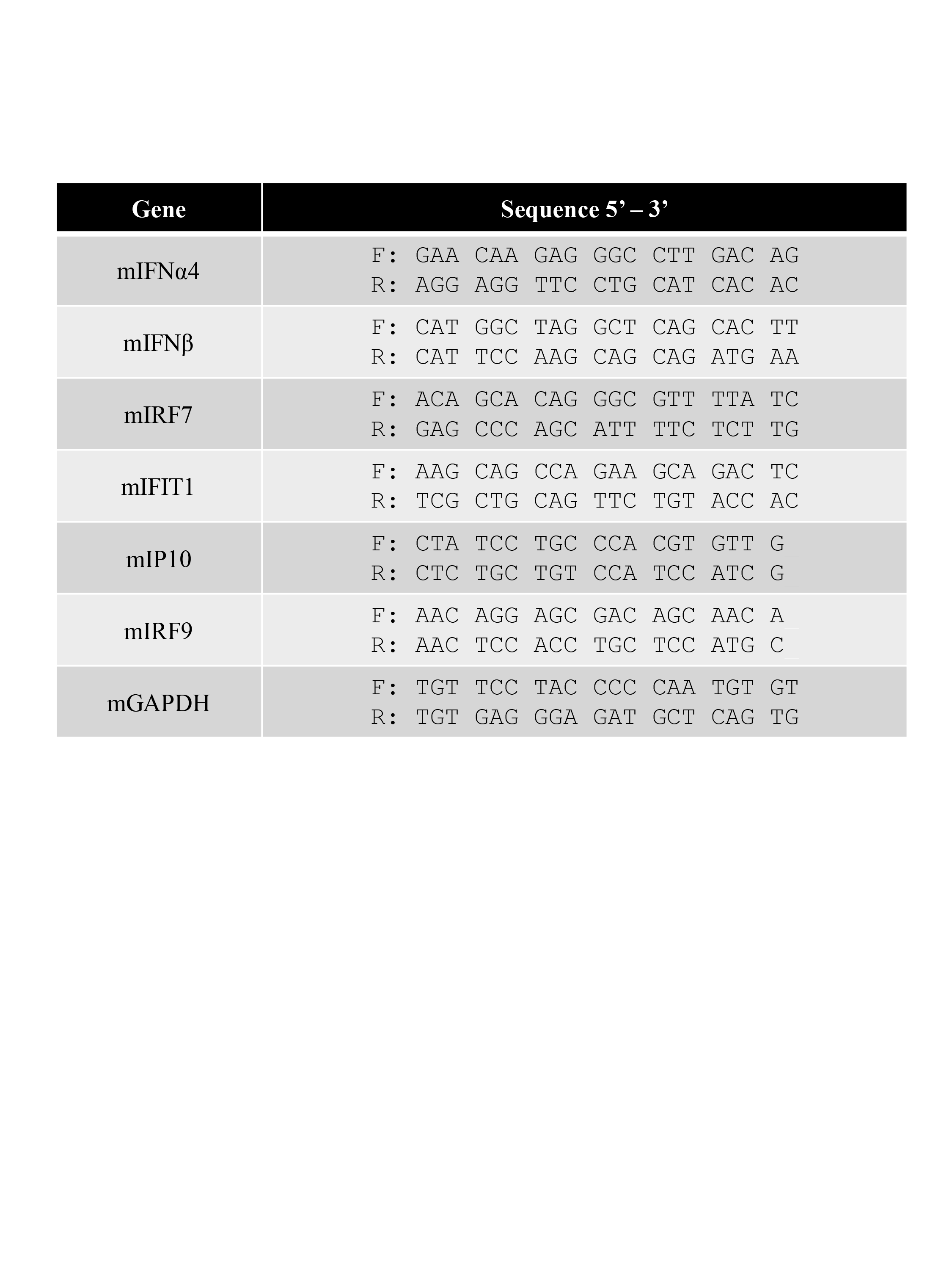

Supplement: Table S1 — RT-PCR primer sequences. (TIF) [file pone.0065801.s005.tif]
